# Adaptive class-aware feature selection for high-dimensional and imbalanced multi-class network intrusion detection

**DOI:** 10.3389/fdata.2026.1871346

**Published:** 2026-06-23

**Authors:** Joseph P. Mchina, Neema Mduma, Ramadhani S. Sinde

**Affiliations:** Computational and Communication Science and Engineering (CoCSE), The Nelson Mandela African Institution of Science and Technology (NM-AIST), Arusha, Tanzania

**Keywords:** adaptive feature selection, machine learning, minority attack detection, mutual information, network intrusion detection systems (NIDS)

## Abstract

High-dimensional feature spaces and severe class imbalance remain fundamental challenges for Machine Learning-based Network Intrusion Detection Systems (ML-NIDS), where minority attack categories are frequently overlooked during feature selection. Existing feature selection approaches commonly rely on global feature relevance measures and manually specified feature counts, which favor majority traffic classes and reduce sensitivity toward rare but critical attack categories. To address these limitations, this study proposes the Adaptive Class-Aware Feature Selection (ACAFS) framework for multi-class intrusion detection. Unlike conventional approaches, ACAFS introduces a data-driven adaptive feature count mechanism based on permutation null hypothesis testing, a Class-Aware Composite Mutual Information scoring strategy that explicitly preserves minority-class discriminative information, and a coordinated two-stage feature selection framework that combines statistical filtering with XGBoost-based model refinement. The framework was evaluated independently on the CSE-CIC-IDS2018 benchmark dataset and a Simulated University Network Environment (SUNE) dataset representing Tanzanian higher learning institution networks. Experimental results demonstrate that ACAFS substantially reduces feature dimensionality while improving balanced intrusion detection performance. On CSE-CIC-IDS2018, ACAFS reduced the feature space from 74 to 22 features, representing a 70.3% dimensionality reduction, while the Two-Stage CNN achieved 99.39% accuracy, 99.40% F1-score, and a false positive rate of 0.09%. The framework further achieved 98.59% recall for Web_Attacks despite severe class imbalance, demonstrating effective preservation of minority-class discriminative features. On the SUNE dataset, ACAFS independently selected 18 features and maintained stable detection performance without dataset-specific manual tuning, confirming its adaptability across heterogeneous network environments. These results confirm that adaptive and class-aware feature selection can simultaneously reduce feature redundancy, improve minority attack detection, and maintain robust intrusion detection performance across diverse network traffic environments.

## 1 Introduction

Despite significant advances in network security, rare but operationally critical attack categories including Web-based attacks and network infiltration continue to evade detection in real-world deployments, causing substantial damage to organizations before they are identified (George et al., 2024). Machine Learning-based Network Intrusion Detection Systems (ML-NIDS) have emerged as a promising response, enabling automated learning of complex traffic patterns and discrimination among diverse attack categories without relying on manually defined signatures (Ngo et al., 2023; Alharbi et al., 2021; Neto et al., 2025). However, two persistent challenges fundamentally limit their effectiveness in practice: high-dimensional feature spaces, where network flow datasets contain large numbers of redundant, correlated, and irrelevant features that increase computational cost and reduce model generalizability (Mauro et al., 2021; Behiry and Aly, 2024; Youssef et al., 2026), and severe class imbalance, where minority attack categories such as Web_Attacks and Infiltration are significantly underrepresented relative to normal traffic, resulting in poor detection sensitivity toward precisely the attack types that are most difficult to identify and most damaging when missed (Nigar and Mustafa, 2025; Mirsadeghi et al., 2023).

Feature selection has therefore emerged as a crucial step in ML-based NIDS, aiming to reduce dimensionality, improve detection performance, and enhance computational efficiency (Bakro et al., 2023; Albulayhi et al., 2022). While class imbalance is commonly addressed using data-level balancing techniques such as SMOTE, existing feature selection methods typically rely on global feature relevance measures that do not explicitly preserve discriminative features for minority attack categories, meaning that important features for rare attacks may be overlooked during the selection process (Zhang et al., 2021; Toleva et al., 2025).

Existing feature selection approaches can be broadly categorized into filter, wrapper, and embedded methods, each presenting inherent trade-offs (Behiry and Aly, 2024; Golrang et al., 2020). Filter methods such as Information Gain, Chi-square, and Mutual Information are computationally efficient but evaluate features independently of the classifier, ignoring inter-feature dependencies and failing to account for class-specific discriminability Min and Wei (2023). For instance, (Korial et al., 2024) demonstrated that integrating Chi-square feature selection with a voting ensemble improved cardiovascular disease detection accuracy by 6.07% and reduced computational load by more than 50%; however, the Chi-square criterion evaluates feature relevance based on the global class distribution and does not account for class-specific discriminability under severe class imbalance, highlighting the continued absence of mechanisms for preserving minority-class discriminative information. Similarly, Dalloo and Humaidi (2024) achieved reductions of 87.5% in computation and 76.9% in memory usage through combined feature selection and sampling strategies, yet their framework does not incorporate class-aware feature scoring to preserve discriminative information for underrepresented categories. Wrapper methods achieve higher predictive performance by evaluating feature subsets using classifier performance but incur prohibitive computational cost for large-scale intrusion detection datasets (Zare and Mahmoudi-Nasr, 2023). Embedded methods integrate feature selection within model training but remain model-dependent and typically rely on fixed parameter configurations that limit adaptability across diverse network environments, contributing to the continued reliance on manually specified or static feature subset selection strategies (Mahanipour and Khamfroush, 2025).

Several studies have explored hybrid and advanced learning-based approaches to overcome these limitations, including particle swarm optimisation, ensemble learning, and hybrid deep learning architectures evaluated on CICIDS2017 and CSE-CIC-IDS2018 (Alzughaibi and El Khediri, 2023; Bakro et al., 2022; Harini et al., 2023; Hu et al., 2024). In the context of CNN-based intrusion detection, (Abed et al., 2024) proposed a modified CNN-IDS model applying PCA and SVD dimensionality reduction combined with Ridge Regression and CNN classifiers on UNSW-NB15, demonstrating that feature selection significantly enhances IDS accuracy and reduces false alarm rates; however, PCA and SVD are global dimensionality reduction techniques that do not explicitly account for minority-class discriminability, and the study does not address the challenge of preserving discriminative features for underrepresented attack categories under severe class imbalance. More broadly, these hybrid approaches primarily emphasize overall detection accuracy while relying on static feature selection techniques that may reduce interpretability and fail to validate feature relevance statistically. Furthermore, many hybrid approaches lack effective coordination between statistical filtering and model-based refinement stages, highlighting the absence of a unified validation mechanism capable of ensuring feature relevance across both statistical and empirical dimensions.

Despite these advances, existing approaches still fail to address three critical and interdependent limitations in feature selection for multi-class intrusion detection. First, most feature selection methods require manual specification of the number of features to select, introducing subjectivity and limiting automation across heterogeneous datasets (Sukhni et al., 2024); for example, approaches proposed by Alzughaibi and El Khediri (2023); Harini et al. (2023) rely on fixed or manually configured feature subsets that are not adaptively determined from the statistical properties of the data, limiting their generalizability across diverse network environments. Second, standard feature scoring techniques such as mutual information evaluate feature relevance based on the overall class distribution, systematically favoring majority classes and failing to preserve discriminative features for minority attack categories (Al-Sarem et al., 2021); in particular, studies such as Abed et al. (2024); Bakro et al. (2022) apply global dimensionality reduction or feature ranking strategies that do not explicitly account for the discriminative requirements of underrepresented attack categories, resulting in reduced sensitivity toward minority classes despite achieving high overall accuracy. Third, existing hybrid approaches lack a coordination mechanism between statistical filtering and model-based refinement, resulting in feature subsets that are not consistently validated across selection stages (Yin et al., 2023; Shi et al., 2025; Pudjihartono et al., 2022); as demonstrated by Hu et al. (2024); Alzughaibi and El Khediri (2023), combining multiple components without a unified validation criterion often produces feature subsets whose relevance is not consistently verified across both statistical and empirical dimensions. Collectively, these three limitations reveal a persistent gap in the existing literature: the absence of a feature selection framework that simultaneously eliminates manual feature count specification, preserves minority-class discriminative information under severe class imbalance, and coordinates statistical filtering with model-based refinement under a unified selection criterion.

While these limitations affect NIDS deployment globally, they are particularly severe in resource-constrained environments. Tanzania's higher learning institutions (HLIs) provide a relevant case, where limited cybersecurity investment and increasing exposure to cyber threats constrain the deployment of effective intrusion detection systems. National evidence indicates that 98% of organizations spend less than USD 5,000 annually on cybersecurity (Mtakati and Sengati, 2021), while studies of Tanzanian HLIs report persistent cyber threats alongside limited cybersecurity infrastructure and expertise (Kifaru et al., 2023; Ntembo and Casmir, 2023; Semlambo and Stanslaus, 2024). These conditions highlight the need for lightweight, adaptive, and scalable intrusion detection frameworks suitable for resource-constrained environments.

To address these limitations simultaneously, this study proposes the Adaptive Class-Aware Feature Selection framework (ACAFS), a statistically adaptive hybrid feature selection pipeline designed for multi-class NIDS. ACAFS introduces three tightly integrated components: a data-driven adaptive feature count mechanism based on permutation null hypothesis testing that derives significance thresholds directly from the statistical properties of the data, eliminating manual feature specification; a Class-Aware Composite Mutual Information scoring function that explicitly incorporates class-specific discriminative information into feature ranking, preserving minority-class representations under severe class imbalance; and a coordinated two-stage selection process that combines statistical filtering with XGBoost-based model refinement under a unified selection criterion, ensuring retained features are validated across both statistical and empirical dimensions. Post-selection class balancing using SMOTE-Tomek is applied exclusively on the selected feature space to reduce synthetic noise. The framework is evaluated independently on the CSE-CIC-IDS2018 benchmark dataset (Thakkar and Lohiya, 2020) and a Simulated University Network Environment (SUNE) representing Tanzanian institutional networks.

The main contributions of this study are as follows:

- A data-driven adaptive feature count mechanism based on permutation null hypothesis testing that automatically identifies statistically significant feature subsets, eliminating manual feature count specification.
- A Class-Aware Composite Mutual Information scoring mechanism that integrates global feature relevance with class-specific discriminative information to improve preservation of minority attack representations under severe class imbalance.
- A coordinated two-stage hybrid feature selection framework that combines statistical filtering with XGBoost-based refinement, ensuring that selected features are both statistically relevant and empirically effective for multi-class intrusion detection.
- A comprehensive independent evaluation on the CSE-CIC-IDS2018 and SUNE datasets, demonstrating substantial dimensionality reduction, strong minority-class detection performance, and consistent cross-dataset generalization without dataset-specific manual tuning.

The remainder of this paper is organized as follows. Section II presents the materials and methods, including dataset description, pre-processing, and the ACAFS framework. Section III reports the experimental results. Section IV discusses the findings and comparisons with existing work. Section V concludes the paper and outlines directions for future research.

## 2 Materials and methods

### 2.1 Datasets

Two datasets were employed for independent evaluation: the CSE-CIC-IDS2018 benchmark dataset and a Simulated University Network Environment (SUNE) dataset developed for this study to reflect traffic conditions typical of higher learning institutions in Tanzania. Both datasets were processed independently through the same pipeline without cross-dataset feature transfer, ensuring consistency across training, validation, and testing partitions.

#### 2.1.1 CSE-CIC-IDS2018 dataset

The CSE-CIC-IDS2018 dataset, developed by the Canadian Institute for Cybersecurity, was created to simulate realistic enterprise network traffic under controlled attack scenarios over a 10-day period (Songma et al., 2023; Thakkar and Lohiya, 2020). The dataset contains approximately 16.1 million network flow records represented by 80 bidirectional flow features extracted using CICFlowMeter v3 across network protocols including HTTP, HTTPS, SSH, SMTP, and POP3.

The traffic generation environment consisted of 50 attacker machines, 420 victim systems, and 30 servers. Malicious traffic was generated using tools such as Hping3, Hydra, Slowloris, and Ares, while benign traffic was produced from simulated user activities across multiple network services (Songma et al., 2023).

For this study, the original 18 traffic subcategories were consolidated into seven main classes: Normal, BruteForce, DoS, Web_Attacks, Infiltration, Botnet, and DDoS to enable consistent multi-class evaluation. The dataset is highly imbalanced, with the Normal class containing 1,169,625 samples compared to only 650 samples for Web_Attacks, resulting in an imbalance ratio of approximately 1,799.4:1. Additional balancing details are provided in Section 2.4.

#### 2.1.2 Simulated university network environment (SUNE) dataset

To complement the CSE-CIC-IDS2018 benchmark, a Simulated University Network Environment (SUNE) dataset was developed to represent network conditions commonly observed in Tanzanian higher learning institutions. Direct acquisition of operational campus traffic was not performed due to institutional privacy and ethical constraints (Noor et al., 2025; Moamin et al., 2025; Njuu et al., 2025). The test environment incorporated Cisco networking devices, a Sophos XGS 2100 firewall, six VLAN segments, and Windows Server 2022 services to emulate a typical institutional network environment.

Cyberattack traffic was generated from a dedicated Kali Linux system and included DoS, Probe, User-to-Root, and Remote-to-Local attack activities using standard penetration testing tools. Normal network activity was generated concurrently across HTTP, HTTPS, SMTP, POP3, DNS, SSH, and FTP services. Traffic collection was conducted over five consecutive days, and labels were assigned based on attack execution timelines. The resulting dataset contains 626,981 flow records described by 45 network traffic features, including 494,664 normal samples and 132,317 attack samples distributed across four attack categories. The evaluation pipeline was applied independently using the native SUNE class structure to preserve dataset-specific traffic characteristics.

### 2.2 Data pre-processing

Prior to feature selection and model training, several pre-processing steps were applied to ensure data quality and prevent information leakage across dataset partitions (Azam et al., 2023; Khraisat and Alazab, 2021). All fitting operations were performed exclusively on the training partition and subsequently applied to the validation and test partitions. The pre-processing pipeline consists of the following steps:

- Non-predictive feature removal: flow identifiers, IP addresses, and timestamp fields were removed, as they do not provide discriminative information for intrusion detection.
- Dataset partitioning: stratified sampling was used to split the dataset into training (70%), validation (15%), and testing (15%) subsets, ensuring class distribution is preserved across all partitions.
- Infinite value handling: infinite values arising from flow-based computations were replaced with missing value indicators to maintain numerical stability.
- Missing value imputation: median imputation was applied using statistics computed from the training partition only, preventing information leakage.
- Feature scaling: numerical attributes were normalized using StandardScaler to produce standardized feature distributions with zero-centered values and unit variance, thereby supporting stable model training and convergence.

### 2.3 Class-aware feature selection framework (ACAFS)

#### 2.3.1 Overview

Effective feature selection in ML-based NIDS requires not only reducing dimensionality but also preserving discriminative information for minority attack categories that are systematically underrepresented in real-world network traffic datasets (Liu et al., 2019; Gul et al., 2025; Albattah and Khan, 2025). To address this challenge, this study proposes the Adaptive Class-Aware Feature Selection framework (ACAFS), a two-stage hybrid pipeline designed to preserve discriminative features for minority attack classes while maintaining overall feature relevance across heterogeneous network environments.

**Figure 1:**
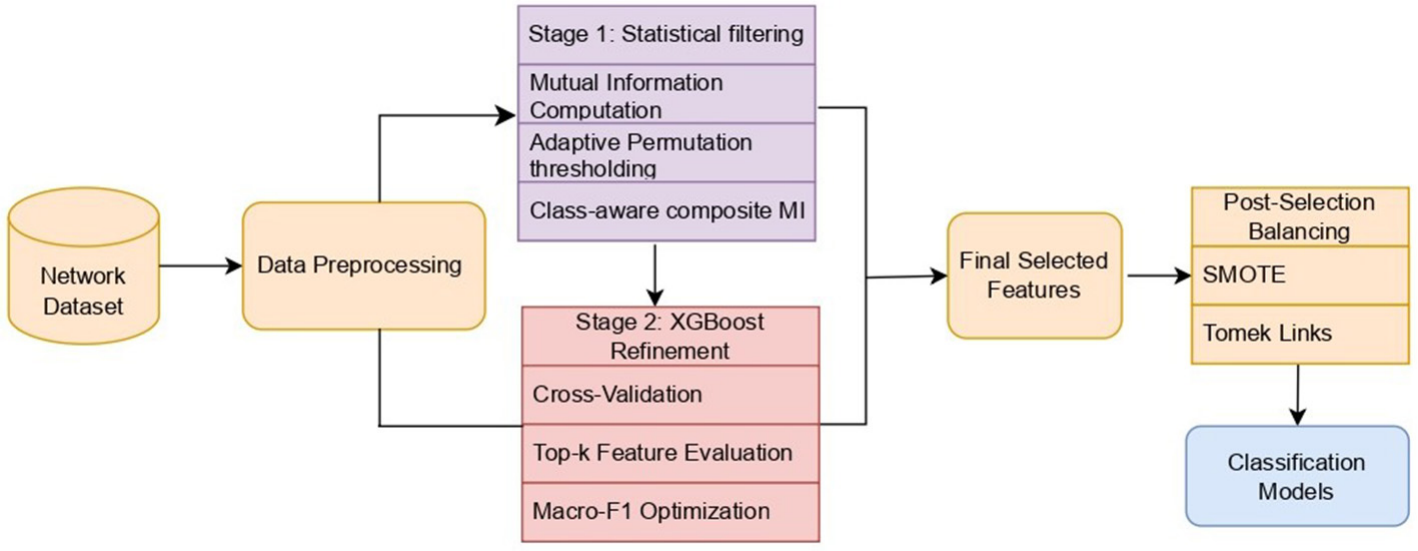
Overview of the proposed ACAFS framework for multi-class intrusion detection.

As illustrated in Figure 1, the ACAFS pipeline operates in two sequential stages. Stage 1 performs statistically grounded filtering by first computing Mutual Information scores to estimate feature relevance. A permutation-based adaptive threshold is then applied to identify statistically significant features. Subsequently, Class-Aware Composite MI scores are derived to ensure that selected features retain discriminative power across all classes, including minority attack categories. In addition, a per-class coverage mechanism is applied to promote balanced representation across attack types and prevent the exclusion of features relevant to low-frequency classes. Stage 2 performs model-based refinement using XGBoost with cross-validation. XGBoost was selected due to its ability to capture non-linear feature interactions and provide reliable feature importance estimates in high-dimensional feature spaces. The optimal feature subset is determined empirically based on classification performance and recall of underrepresented attack categories. Only features that satisfy both statistical significance in Stage 1 and empirical predictive effectiveness in Stage 2 are retained in the final subset.

This dual-evidence selection strategy provides a coordinated and adaptive feature selection mechanism, addressing limitations of existing hybrid approaches that combine statistical filtering and model-based refinement without a unified selection criterion. As a result, the framework improves the preservation of minority-class discriminative features while maintaining overall model performance. The framework was applied independently to both datasets, reducing the feature space while preserving discriminative capability across both majority and minority attack categories.

#### 2.3.2 Class-aware composite mutual information

Mutual Information (MI) quantifies the statistical dependence between a feature and the target variable by measuring the reduction in uncertainty about the class label given knowledge of the feature value. For a feature X and class label Y, the standard multiclass MI is formally defined as Papaioannou et al. (2025); Leiva-Murillo and Artes-Rodriguez (2007).


$$\begin{array}{l} MI\left(X,Y\right)=\underset{x\epsilon X}{\sum }\underset{y\epsilon Y}{\sum }p\left(x,y\right)\log\frac{p\left(x,y\right)}{p\left(x\right)p\left(y\right)} \end{array}$$ (1)

where *p*(*x, y*) is the joint probability distribution and *p*(*x*), *p*(*y*) are the respective marginal distributions.

Existing global MI evaluates feature relevance with respect to the overall class distribution, which inherently assigns greater weight to majority classes (Gul et al., 2025; Albattah and Khan, 2025). In severely imbalanced multi-class datasets such as CSE-CIC-IDS2018, this leads to the selection of features that are informative for dominant classes while failing to preserve features that are discriminative for low-frequency attack types such as Web_Attacks (Chen et al., 2024).

To address this limitation, the proposed ACAFS framework introduces a Class-Aware Composite MI scoring formulation that explicitly incorporates minority-class discriminability into the feature ranking process. For each class *cϵC*, a One-vs.-Rest MI score is computed following class-specific relevance estimation strategies commonly used in multi-class feature evaluation (Al-Sarem et al., 2021; Gul et al., 2025). Within the proposed framework, the Composite MI score for each feature *f* is then defined as:


$$\begin{array}{c} COMPOSITE\;\left(f\right)=\;\alpha \;\times MI\text{\_}global\left(f\right)+\left(1-\alpha \right)\\ \;\times min\text{\_}c\;\left(MI_{OvR\left(f,c\right)}\right) \end{array}$$ (2)

where *MI*_*global*(*f*) is the standard multiclass MI score, *min*_*c*(*MI*_*OvR*(*f, c*)) represents the worst-case class-specific relevance of the feature. In this study, the parameter α = 0.6 was selected based on both theoretical reasoning and empirical validation. In severely imbalanced datasets, global MI scores are inherently dominated by majority-class statistics, potentially underweighting features that are discriminative for minority attack categories. The selected value assigns 60% of the weight to global feature relevance and 40% to minority-class discriminability, providing a balance between overall feature utility and minority-class preservation. The sensitivity analysis presented in Section 3.3 further supports this choice.

To further ensure representation of all attack categories, a per-class coverage mechanism is introduced. Motivated by information-theoretic principles, where distinguishing among *C* discrete classes requires approximately log_2_(*C*) bits of information (Cover and Thomas, 2006), the minimum number of features guaranteed for each class is defined as:


$$\begin{array}{l} MIN\text{\_}PER\text{\_}CLASS=\left[\text{lo}{\text{g}}_{2}\left(C\right)\right] \end{array}$$ (3)

where *C* is the number of classes. For *C* = 7 classes, this yields a minimum of 3 features per class. This formulation provides an information-theoretic lower bound for per-class feature coverage. Compared with linear scaling, which may introduce redundant features as the number of classes increases, and constant minimums, which do not adapt across datasets, the logarithmic formulation offers a scalable compromise between minority-class preservation and feature compactness.

#### 2.3.3 Permutation-based adaptive threshold

To determine which features contain statistically significant information rather than random noise, ACAFS employs a permutation-based null hypothesis mechanism that derives a dataset-specific significance threshold directly from the data. The null distribution of MI scores is constructed by randomly permuting the class labels 100 times and recomputing MI for each permutation. This process breaks the true association between features and labels while preserving marginal distributions, producing MI values expected under the null hypothesis of no association. The use of 100 permutations provides a computationally efficient approximation of the null distribution for large-scale network traffic datasets. Following the permutation-based mutual information feature selection framework of François et al. (2007), this study generates a null distribution of mutual information (MI) scores through random permutation of class labels and defines the adaptive significance threshold τ as:


$$\begin{array}{l} \tau =Quantile_{0.99}\left(MI\text{\_}null\right) \end{array}$$ (4)

where *MI*_*null*_ represents the distribution of MI scores obtained under label permutation.

This threshold adapts to the statistical properties of each dataset, eliminating the need for manual specification. Features with global MI scores exceeding τ are retained as statistically significant candidates.

Following thresholding, features are ranked using Composite MI scores, and the MIN_PER_CLASS coverage constraint is applied to ensure representation across all attack categories. A maximum cap is then imposed to define the Stage 1 candidate feature pool, which is subsequently passed to Stage 2 for model-based refinement.

#### 2.3.4 XGBoost-based refinement

Stage 2 performs model-based refinement to determine the optimal feature count from the Stage 1 candidate pool. An XGBoost classifier is trained and evaluated using stratified cross-validation across a range of candidate feature counts. For each candidate k, the top-k features ranked by Composite MI score are evaluated, and the macro-averaged F1-score along with per-class recall for minority attack categories are recorded. To optimize this trade-off between overall performance and minority-class sensitivity, the proposed ACAFS framework defines the optimal feature count k^*^ is defined as:


$$\begin{array}{l} k^{*}=argmax_{k}\;[F1_{macro\left(k\right)}-w_{std}\times Std\left(F1\left(k\right)\right)+w_{web}\\ \;\times Recall_{web\left(k\right)}+w_{\inf}\times Recall_{\inf\left(k\right)}] \end{array}$$ (5)

Subject to: *F*1_*macro*_(*K*) ≥ *F*1_*best*_ − ϵ

where ϵ is a small tolerance margin defining the admissible performance range, *w*_*std*_ penalizes instability across folds, and *w*_*web*_ and *w*_inf_ assign equal weighting to the recall of underrepresented attack categories. In this study, all weights were set equally to *w*_*std*_ = *w*_*web*_ = *w*_inf_ = 1 to avoid bias toward any specific component and to ensure balanced consideration of classification stability, Web_Attacks recall, and Infiltration recall during feature subset selection. The tolerance parameter was set to ε = 0.01, allowing candidate feature subsets whose macro F1-score remained within 1% of the best observed performance.

**Figure 2:**
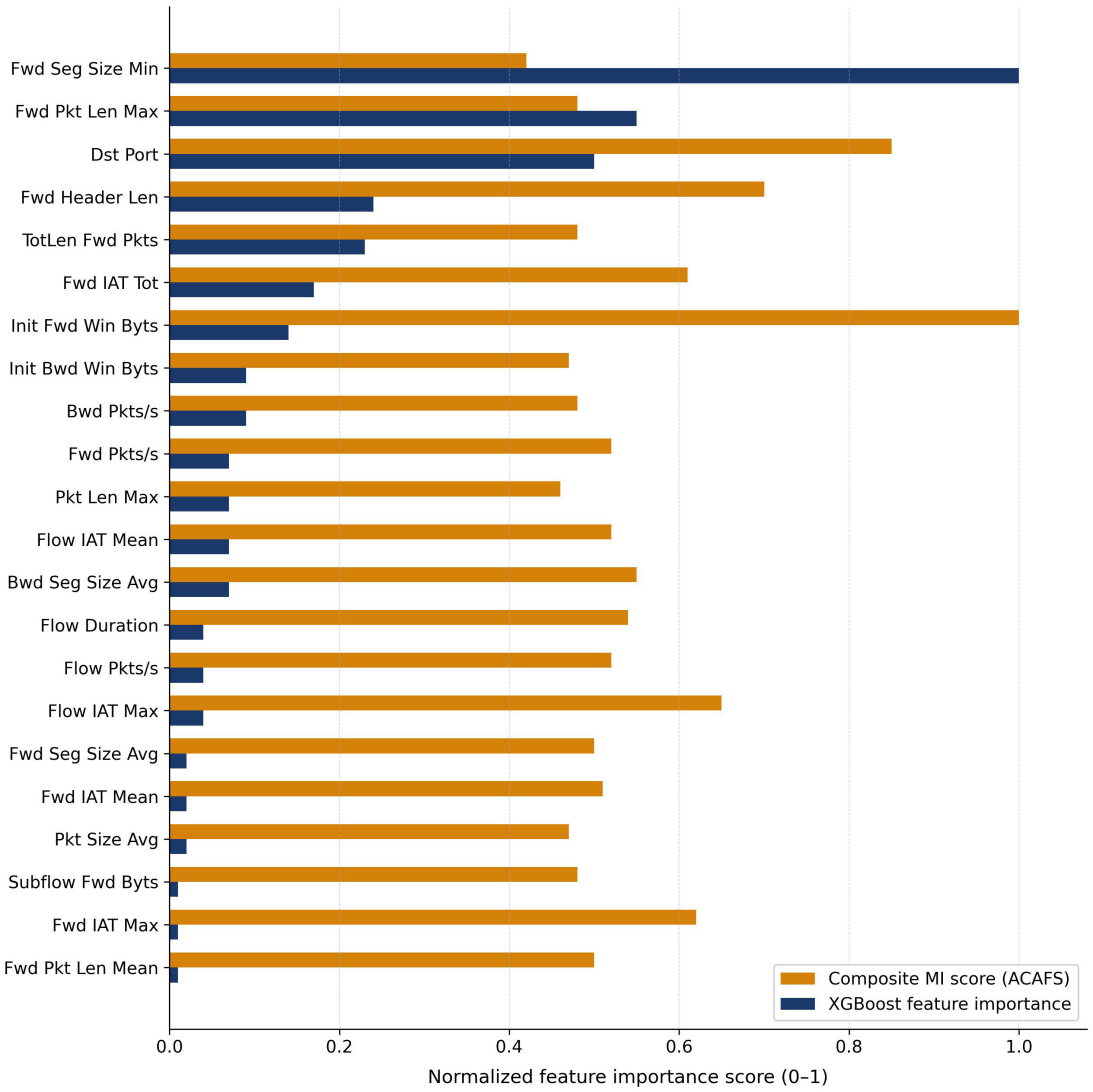
Feature importance and Composite MI scores generated by ACAFS on the CSE-CIC-IDS2018 dataset.

The framework selected 22 features for CSE-CIC-IDS2018 and 18 features for SUNE, reflecting dataset-specific characteristics rather than inconsistencies in the selection process. The two stages of ACAFS rely on complementary sources of evidence: stage 1 evaluates statistical feature relevance, while Stage 2 validates empirical predictive performance. This dual validation strategy ensures that the selected feature subset is both statistically meaningful and practically effective. The final selected feature subset for CSE-CIC-IDS2018 is reported in Table 1, while Figure 2 illustrates the corresponding Composite MI and XGBoost importance scores used during the two-stage selection process.

**Table 1:** Final feature subset selected by ACAFS for CSE-CIC-IDS2018.

Rank	Selected feature	Rank	Selected feature	Rank	Selected feature
1	Fwd seg size min	9	Bwd Pkts/s	17	Fwd seg size avg
2	Fwd Pkt Len max	10	Fwd Pkts/s	18	Fwd IAT mean
3	Dst port	11	Pkt Len max	19	Pkt size avg
4	Fwd header len	12	Flow IAT mean	20	Subflow Fwd Byts
5	TotLen fwd pkts	13	Bwd seg size avg	21	Fwd IAT max
6	Fwd IAT tot	14	Flow duration	22	Fwd Pkt Len mean
7	Init Fwd Win Byts	15	Flow Pkts/s		
8	Init Bwd Win Byts	16	Flow IAT max		

### 2.4 Class balancing

The CSE-CIC-IDS2018 training partition exhibits severe class imbalance, with the Normal class dominating while critical attack categories such as Web_Attacks and Infiltration remain underrepresented. To address this issue, class balancing was applied exclusively to the training partition after feature selection, ensuring that synthetic samples are generated within a reduced feature space. Validation and test partitions were left unchanged to preserve unbiased performance evaluation.

A multi-stage SMOTE-Tomek strategy (Chawla et al., 2002; Yousefimehr et al., 2025) was employed. First, pre-undersampling was applied to cap the Normal and DDoS classes at 150,000 and 80,000 samples, respectively. Next, SMOTE with *k* = 5 nearest neighbors was used to increase minority class representation, targeting 25,000 samples for BruteForce, DoS, and Botnet, 20,000 for Web_Attacks, and 30,000 for Infiltration. Finally, Tomek Links cleaning was applied to remove borderline overlapping samples, improving class separability. The balancing procedure was also applied independently to the SUNE training partition. Table 2 presents the class distribution before and after class balancing.

**Table 2:** Class distribution before and after class balancing for CSE-CIC-IDS2018.

Class	Before balancing	After-SMOTE-Tomek
Normal	1,169,625	141,653
BruteForce	33,274	33,272
DoS	57,151	57,150
Web_Attacks	650	20,000
Infiltration	31,500	23,169
Botnet	24,998	25,000
DDoS	110,403	80,000

### 2.5 Classification models

Five classification models representing diverse learning paradigms were employed to evaluate the effectiveness of the ACAFS feature selection framework. The selection includes deep learning, transformer-based, and ensemble models to assess whether the selected features generalize across different model architectures.

To isolate the contribution of feature selection, each model was evaluated under two conditions: using all pre-processed features and using only the ACAFS-selected subset. All models were trained on the SMOTE-Tomek balanced training partition and evaluated on the original unbalanced test partition to reflect realistic deployment conditions. Model performance was assessed using accuracy, precision, recall, macro-averaged F1-score, and false positive rate, as described in Section 2.6. The same models and configurations were applied independently to both CSE-CIC-IDS2018 and SUNE datasets.

#### 2.5.1 Two-stage convolutional neural network

A Two-Stage CNN was employed as the primary deep learning classifier. Its hierarchical design a Gate CNN for binary normal/attack separation followed by an Attack CNN for fine-grained attack classification was selected for its ability to reduce class competition during optimization and improve sensitivity toward minority attack categories. Temperature scaling was applied after training to improve probability calibration (Balanya et al., 2024).

#### 2.5.2 TabTransformer

TabTransformer was included to evaluate whether ACAFS-selected features support effective attention-based feature interaction learning in tabular intrusion detection data (Huang et al., 2020).

#### 2.5.3 XGBoost

XGBoost was selected as an ensemble baseline due to its strong performance on high-dimensional tabular data and its role as the Stage 2 refinement model within the ACAFS framework, enabling direct comparison between its standalone and feature-selection-guided performance (Chen and Guestrin, 2016).

#### 2.5.4 Random forest

Random Forest was included as a tree-based ensemble baseline to assess the generalization of ACAFS-selected features across a model architecture that is independent of the Stage 2 XGBoost refinement process (Cutler et al., 2012).

#### 2.5.5 LightGBM

LightGBM was included to assess whether the ACAFS-selected feature subset supports efficient gradient boosting performance under the severe class imbalance conditions present in both evaluation datasets (Li et al., 2024).

### 2.6 Evaluation metrics

Each classifier was evaluated using five standard metrics derived from the confusion matrix: accuracy, precision, recall, macro-averaged F1-score, and false positive rate (FPR) (Owusu-Adjei et al., 2023). Given the severe class imbalance in both evaluation datasets, macro-averaged F1-score was adopted as the primary evaluation metric, as it assigns equal weight to all classes including minority attack categories such as Web_Attacks and Infiltration, and is therefore more informative than overall accuracy alone. FPR was additionally reported to assess false alarm control, which is a critical operational requirement for real-world NIDS deployment.

### 2.7 Implementation details

The experimental workflow was implemented in Google Colab using Python 3.12 on a Linux x86_64 runtime with 12 GB RAM. Model training and evaluation used Scikit-learn 1.6.1, XGBoost 3.0.5, LightGBM, imbalanced-learn 0.14.0, and PyTorch, while NumPy, Pandas, and Matplotlib supported data handling and visualization. To improve reproducibility, all experiments were executed with a fixed random seed of 42. The Two-Stage CNN was trained using the Adam optimizer with temperature scaling applied after training for probability calibration, while the TabTransformer was trained using AdamW with early stopping to prevent overfitting. XGBoost was configured using standard gradient boosting settings, and Random Forest and LightGBM were trained using default configurations. For the ACAFS framework, the Composite Mutual Information weighting parameter was set to α = 0.6, and the permutation-based null distribution was constructed using 100 permutations, with a bounded search strategy and a small admissibility tolerance ε applied in the second stage to ensure stable feature subset selection.

## 3 Results

### 3.1 ACAFS feature selection results

The ACAFS framework was applied independently to both datasets. For CSE-CIC-IDS2018, the permutation-based adaptive threshold was determined as τ ≈ 0.0069 using 100 label permutations and the 99th percentile of the null distribution. Of the 74 candidate features, 65 exceeded this threshold and were retained as statistically significant. Following Composite MI scoring and per-class coverage enforcement, a candidate pool of 40 features was passed to Stage 2. Stage 2 cross-validation subsequently selected a final subset of 22 features, representing a 70.3% reduction in dimensionality. The highest-ranked feature was *Init Fwd Win Byts* with a Composite MI score of 1.0000. Figure 2 presents the normalized XGBoost importance scores alongside Composite MI scores for the 22 selected features, showing general alignment between Stage 1 statistical ranking and Stage 2 model-based refinement. For reproducibility, the complete set of 22 features selected by ACAFS for CSE-CIC-IDS2018 is reported in Table 1. The selected subset includes temporal flow characteristics, packet-level statistics, traffic rate measures, and protocol-related attributes, providing a compact yet discriminative representation of network behavior for intrusion detection.

For SUNE, the same pipeline produced an adaptive threshold of τ ≈ 0.0039, reflecting the distinct statistical characteristics of university network traffic, and selected 18 features representing a 60% dimensionality reduction. The top-ranked feature was *Connection duration* with a Composite MI score of 1.0000. The substantial dimensionality reduction achieved across both datasets further suggests that many original flow features contributed limited additional discriminative information.

### 3.2 Ablation study of ACAFS stages

To assess the contribution of each ACAFS stage, an ablation study was conducted on CSE-CIC-IDS2018 using the Two-Stage CNN under identical experimental conditions. Three configurations were evaluated: (i) Global MI-only, which ranks features using standard global Mutual Information; (ii) Stage 1, which incorporates adaptive thresholding, Class-Aware Composite MI scoring, and MIN_PER_CLASS coverage enforcement; and (iii) Full ACAFS, which further applies Stage 2 XGBoost-based refinement. The performance comparison is presented in Table 3.

**Table 3:** Ablation study of ACAFS stages on CSE-CIC-IDS2018 using the Two-Stage CNN.

Feature selection	No. of features	Accuracy	Precision	Recall	F1-Score	FPR	Web_Recall	Inf_Recall
Global MI only	31	97.42	97.10	97.42	96.88	1.84	91.37	94.85
Stage 1 only	40	98.76	98.81	98.76	98.63	0.94	96.85	97.94
Full ACAFS	22	99.39	99.43	99.39	99.40	0.09	98.59	98.69

The results show a consistent improvement across successive ACAFS stages. Compared with Global MI-only, Stage 1 improved minority-class detection, increasing Web_Attacks recall from 91.37 to 96.85% and Infiltration recall from 94.85 to 97.94%, while reducing FPR from 1.84 to 0.94%. Applying Stage 2 refinement further reduced the feature subset from 40 to 22 features and achieved the best overall performance, including a 99.40% F1-score and 0.09% FPR. These findings demonstrate that Stage 1 enhances minority-class feature preservation, while Stage 2 removes redundant features and improves overall classification performance.

### 3.3 Sensitivity analysis of ACAFS parameters

The results in Table 4 indicate that ACAFS maintains stable performance across all tested α values, with accuracy varying between 99.36 and 99.39% and F1-score varying between 99.37 and 99.40%. The highest F1-score (99.40%), lowest FPR (0.090%), and strongest overall minority-class detection performance were achieved at α = 0.6, which was therefore adopted in the proposed framework. The results in Table 5 show that performance improves slightly as the number of permutations increases and stabilizes from 100 permutations onward. Beyond this point, both the selected feature count and classification metrics remained nearly unchanged. Consequently, 100 permutations were selected as a computationally efficient operating configuration for all experiments.

**Table 4:** Sensitivity analysis of α in the ACAFS framework on CSE-CIC-IDS2018.

α	No. of Features	Accuracy	F1-Score	FPR	Web_Recall	Inf_Recall
0.2	23	99.36	99.37	0.094	98.11	98.67
0.4	22	99.36	99.37	0.094	98.34	98.77
0.6	22	99.39	99.40	0.090	98.56	98.59
0.8	23	99.37	99.38	0.093	98.29	98.62
1.0	23	99.37	99.38	0.092	98.94	98.37

**Table 5:** Sensitivity analysis of the permutation count in the ACAFS framework on CSE-CIC-IDS2018.

No. of permutations	No. of features	Accuracy	F1-score	FPR	Web_Recall	Inf_Recall
25	24	99.32	99.33	0.098	98.21	98.08
50	23	99.36	99.37	0.094	98.43	98.34
100	22	99.39	99.40	0.090	98.56	98.59
150	22	99.39	99.40	0.089	98.59	98.61
200	22	99.39	99.40	0.089	98.59	98.63

### 3.4 Stability analysis across random seeds

To evaluate the reproducibility of ACAFS, the complete pipeline was executed using five different random seeds. The results are summarized in Table 6. The selected feature count varied only between 21 and 22 features, while accuracy and F1-score exhibited standard deviations of only 0.02%. Similarly, FPR showed a standard deviation of 0.01%, and minority-class recall remained highly consistent across all runs. These findings indicate that ACAFS produces stable feature subsets and classification performance that are not dependent on a specific random initialization.

**Table 6:** Stability analysis of ACAFS across five random seeds on CSE-CIC-IDS2018.

Seed	Feature	Accuracy	F1-score	FPR	Web_Recall	Inf_Recall
0	21	99.35	99.36	0.11	98.12	98.28
7	22	99.37	99.38	0.10	98.31	99.81
42	22	99.39	99.40	0.09	98.56	98.59
123	21	99.38	99.39	0.09	98.34	98.73
2024	22	99.38	99.38	0.10	98.47	98.81
Mean	21.6	99.37	99.38	0.10	98.36	98.64
Std	0.55	0.02	0.02	0.01	0.17	0.22

### 3.5 Classification performance on CSE-CIC-IDS2018

Table 7 presents the classification performance of all five models on CSE-CIC-IDS2018 using ACAFS-selected features. Deep learning models consistently achieved the strongest overall performance, with the Two-Stage CNN obtaining an accuracy of 99.39%, F1-score of 99.40%, and the lowest FPR of 0.09%. TabTransformer achieved the second-highest F1-score at 98.96%, while XGBoost, Random Forest, and LightGBM achieved F1-scores of 96.90, 96.89, and 93.85%, respectively. The consistently strong performance observed across diverse model architectures suggests that ACAFS retained informative and discriminative features while reducing redundant dimensions.

**Table 7:** Classification performance on CSE-CIC-IDS2018 with ACAFS feature selection.

Classifier	Accuracy	Precision	Recall	F1-score	FPR
CNN	99.39	99.43	99.39	99.40	0.009
TabTransformer	98.85	98.97	98.85	98.96	0.0016
XGBoost	97.31	97.10	97.31	96.90	0.0148
Random Forest	97.25	97.10	97.25	96.89	0.0152
LightGBM	96.59	94.20	96.59	93.85	0.0130

Table 8 presents classification performance without ACAFS feature selection. All models showed performance degradation when evaluated using the full feature space. The largest decline was observed for XGBoost, whose F1-score decreased from 96.90 to 83.93%, accompanied by an increase in FPR from 1.48 to 3.12%. TabTransformer showed the smallest decline, with F1-score decreasing marginally from 98.96 to 98.79%. Random Forest and the Two-Stage CNN also exhibited reduced performance compared to the ACAFS-selected feature subset. These results suggest that ACAFS improved feature relevance while reducing the negative impact of redundant and noisy dimensions across multiple learning paradigms.

**Table 8:** Classification Performance on CSE-CIC-IDS2018 without ACAFS.

Classifier	Accuracy	Precision	Recall	F1-score	FPR
CNN	97.06	96.90	97.06	96.87	0.0044
TabTransformer	98.62	98.80	98.62	98.79	0.0019
XGBoost	84.56	85.20	84.56	83.93	0.0312
Random Forest	88.10	91.70	88.10	91.56	0.0235
LightGBM	91.63	93.90	91.63	93.85	0.0155

**Figure 3:**
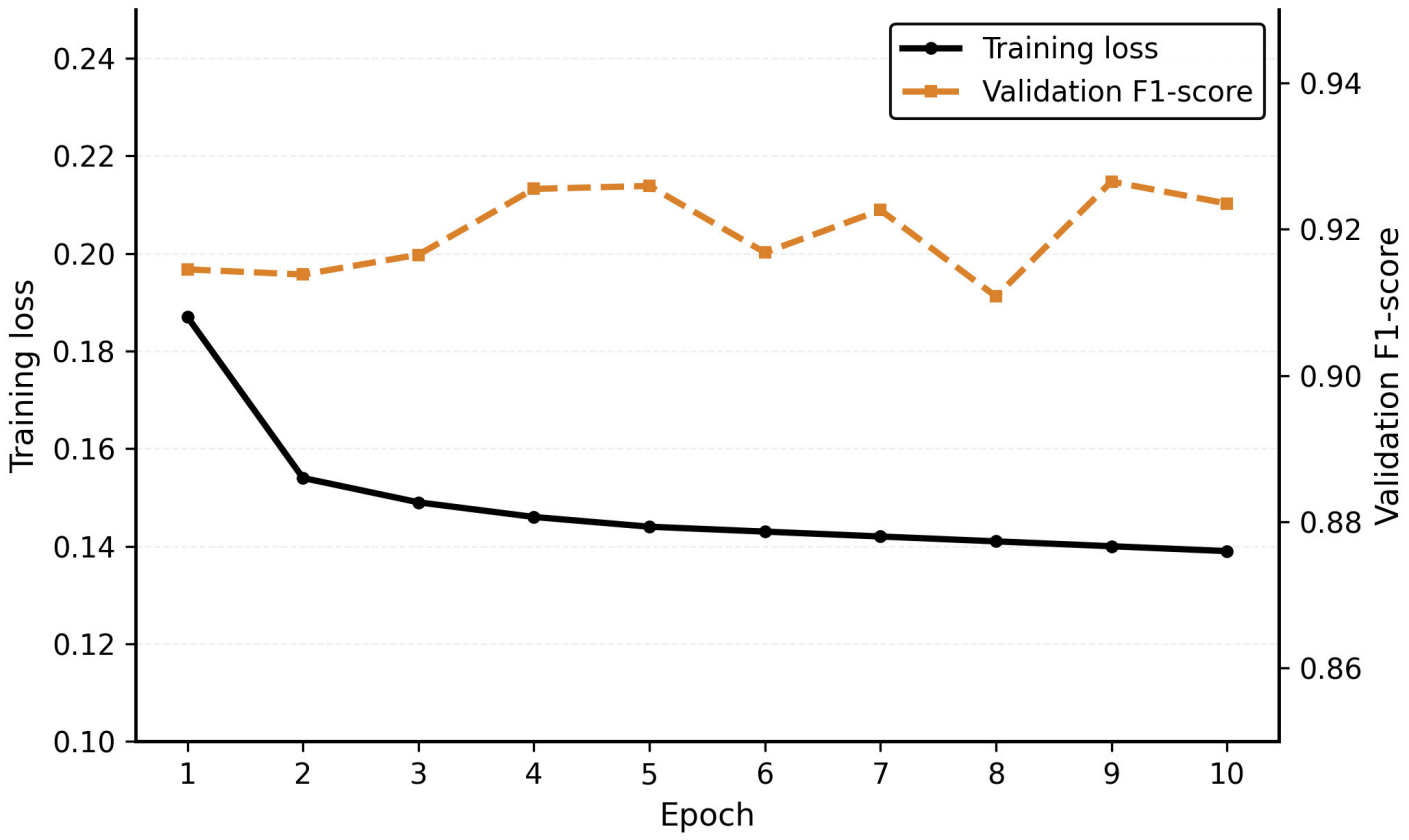
Training loss and validation macro F1-score curves for the Gate CNN on CSE-CIC-IDS2018.

**Figure 4:**
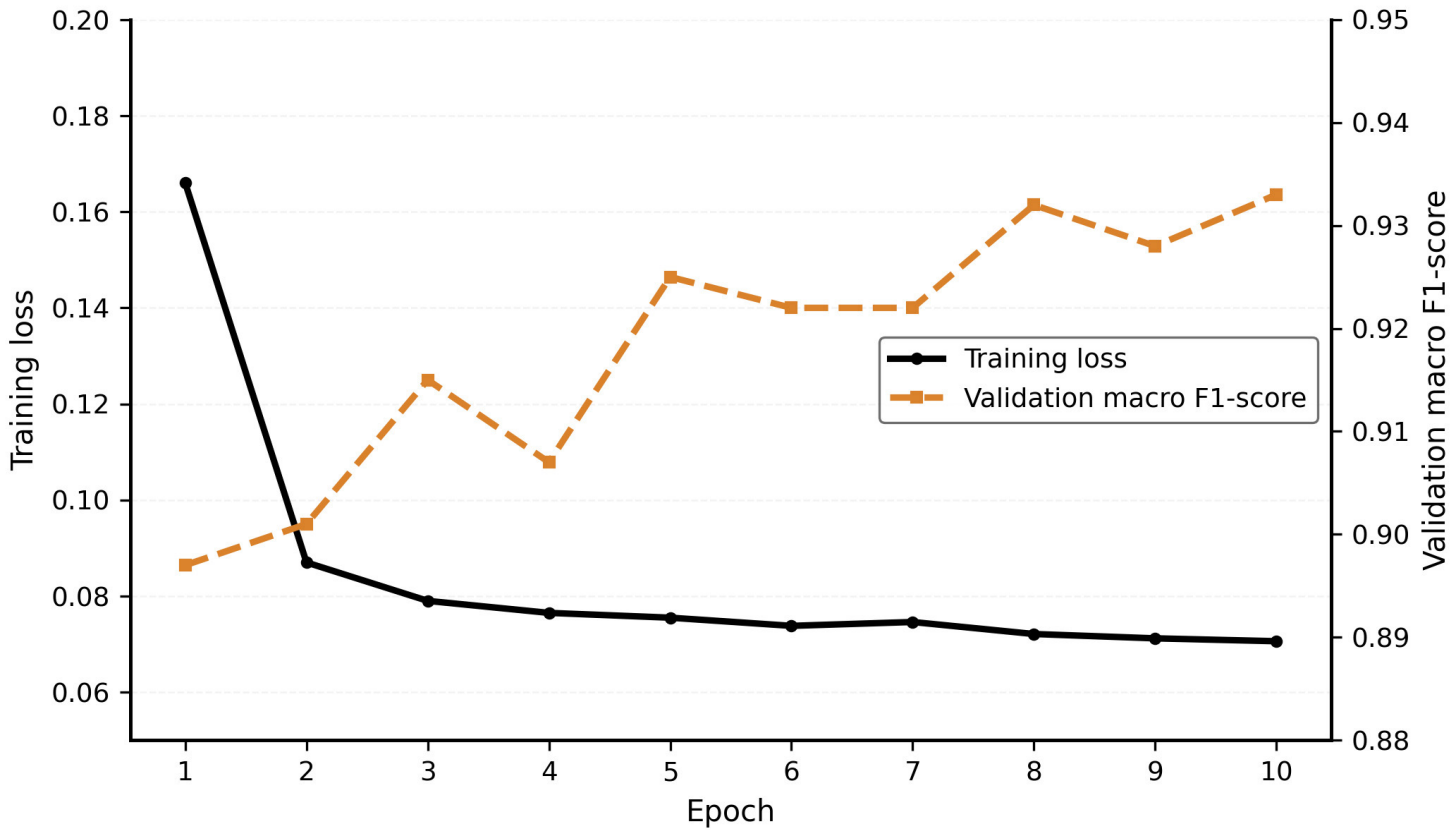
Training loss and validation macro F1-score curves for the Attack CNN on CSE-CIC-IDS2018.

Figures 3, 4 present the training loss and validation F1-score curves for the Gate CNN and Attack CNN, respectively, across 10 training epochs on CSE-CIC-IDS2018. Both stages demonstrated stable convergence with progressively decreasing training loss. The Attack CNN showed a gradual increase in validation macro F1-score, reaching 0.930 at epoch 10 without clear evidence of overfitting.

**Figure 5:**
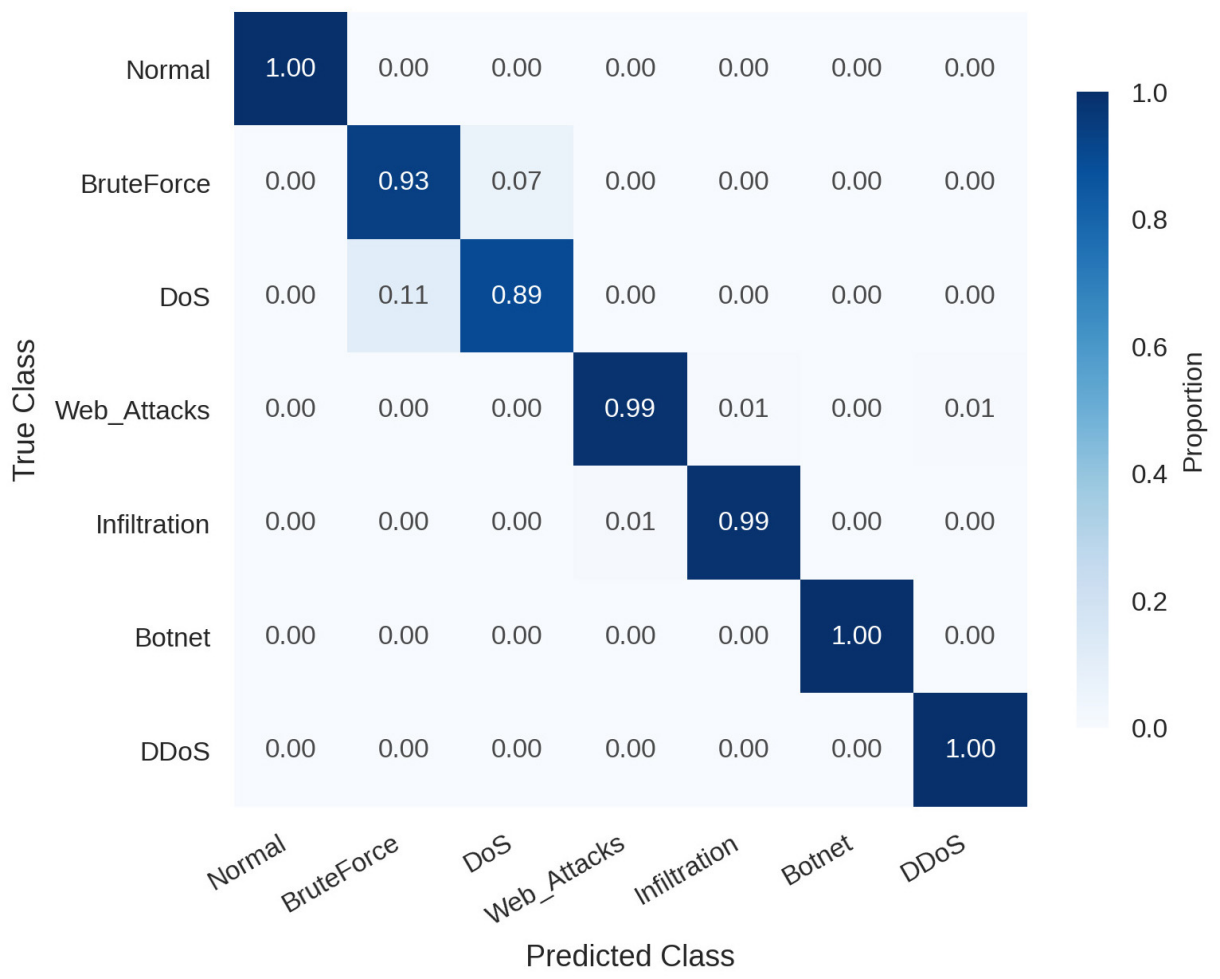
Normalized confusion matrix for the Two-Stage CNN on CSE-CIC-IDS2018.

Table 9 presents the per-class performance of the Two-Stage CNN on CSE-CIC-IDS2018. Strong performance was observed across all seven traffic categories, including minority attack classes. Web_Attacks achieved a recall of 98.56% despite representing 0.045% of the test set, suggesting that ACAFS preserved discriminative information relevant to this minority class. However, the comparatively lower precision of 63.43% indicates that overlap between Web_Attacks and other traffic patterns still generated false positives. Infiltration achieved an F1-score of 99.26% with a recall of 98.59%, while DDoS, Botnet, and Normal traffic achieved F1-scores exceeding 99.90%. BruteForce and DoS achieved F1-scores of 88.30 and 92.55%, respectively. Figure 5 presents the normalized confusion matrix for the Two-Stage CNN on CSE-CIC-IDS2018, showing strong diagonal dominance and limited inter-class confusion.

**Table 9:** Per-class performance of the Two-Stage CNN on CSE-CIC-IDS2018.

Class	Precision	Recall	F1-score	Support
Normal	0.9996	1.0000	0.9998	250,634
Brute Force	0.8372	0.9341	0.8830	7,131
DoS	0.9588	0.8945	0.9255	12,247
Web_Attacks	0.6343	0.9859	0.7718	139
Infiltration	0.9994	0.9859	0.9926	6,750
Botnet	0.9985	0.9996	0.9991	5,356
DDoS	0.9997	0.9999	0.9998	23,658

### 3.6 Classification performance on SUNE

Table 10 presents the classification performance on SUNE using ACAFS-selected features. The Two-Stage CNN achieved the highest overall performance with an accuracy of 96.48% and weighted F1-score of 95.77%, while Random Forest achieved the lowest FPR at 0.49%. XGBoost and LightGBM achieved comparable F1-scores of 95.40 and 95.48%, respectively. TabTransformer achieved the lowest F1-score at 89.90%, indicating comparatively lower generalization performance on the SUNE dataset. Despite the increased variability and institutional specificity of SUNE traffic, all models maintained relatively low FPR values, supporting the practical suitability of the selected features for realistic deployment scenarios.

**Table 10:** Classification performance on SUNE with ACAFS feature selection.

Classifier	Accuracy	Precision	Recall	F1-score	FPR
CNN	96.48	97.18	96.17	95.77	0.0081
TabTransformer	91.93	90.26	90.32	89.00	0.0168
XGBoost	95.90	95.89	96.20	95.90	0.0089
Random Forest	94.34	95.95	94.34	93.99	0.0049
LightGBM	95.76	95.14	95.76	95.76	0.0091

**Figure 6:**
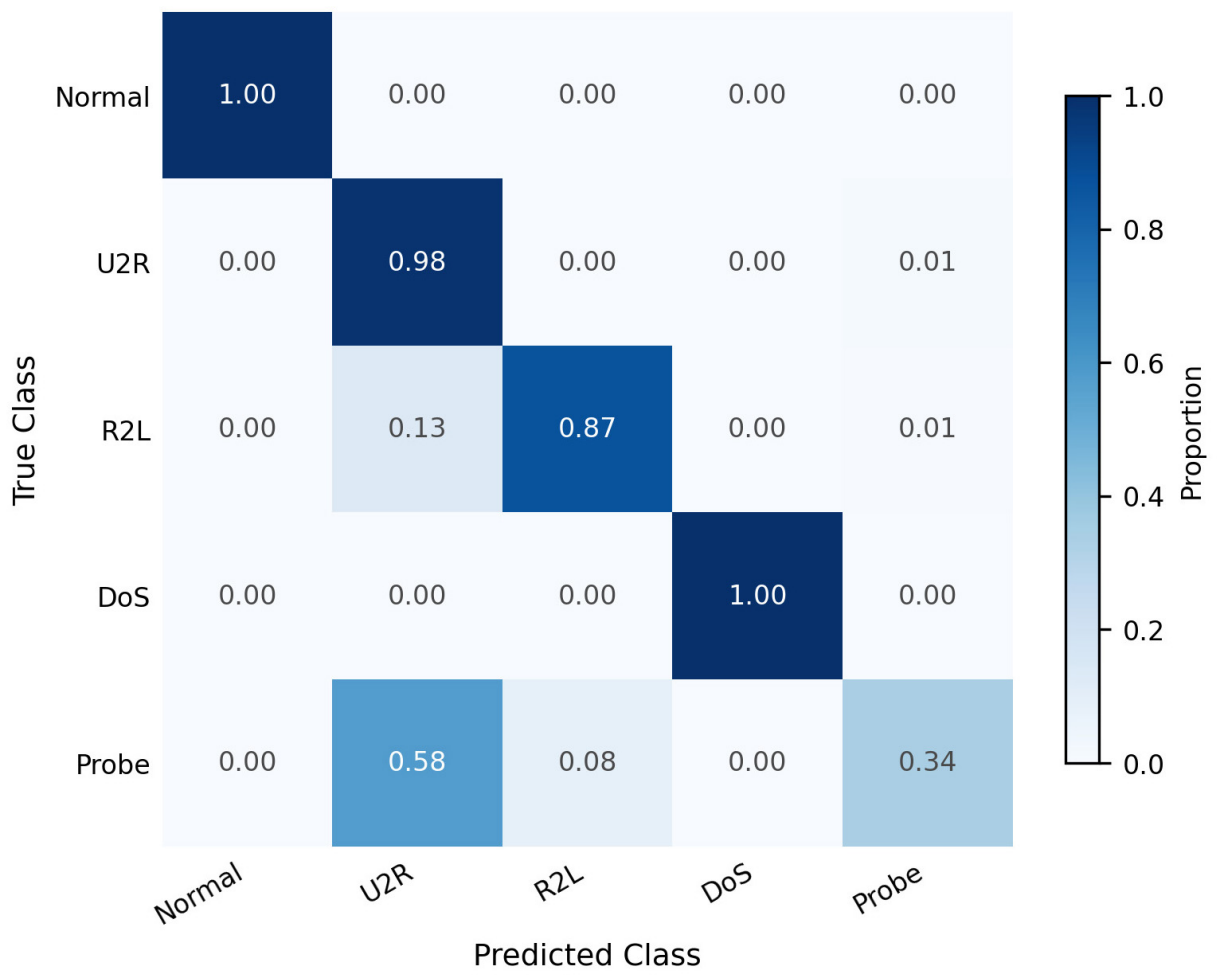
Normalized confusion matrix for the Two-Stage CNN on the SUNE dataset.

Table 11 presents the per-class performance on SUNE. Normal and DoS traffic achieved near-perfect classification with F1-scores approaching 100%. R2L achieved an F1-score of 88.43%, while U2R achieved 76.39%. Probe achieved the lowest recall at 33.90% and an F1-score of 49.84%, indicating comparatively higher classification difficulty for reconnaissance-based traffic patterns. This may reflect behavioral similarity between Probe traffic and legitimate network scanning activity, reducing class separability within the feature space. Figure 6 presents the normalized confusion matrix for the Two-Stage CNN on SUNE, showing strong diagonal dominance across most classes, with Probe exhibiting the largest degree of confusion.

**Table 11:** Per class performances on SUNE.

Class	Precision	Recall	F1-score	Support
Normal	0.9995	1.0000	0.9997	56,769
U2R	0.6263	0.9753	0.7628	4,088
R2L	0.9034	0.8682	0.8854	3,187
DoS	1.000	1.0000	1.0000	5,468
Probe	0.9145	0.3496	0.5058	3,487

### 3.7 Cross-dataset consistency analysis

The performance comparison across CSE-CIC-IDS2018 and SUNE demonstrates consistent detection capability across heterogeneous network environments. The Two-Stage CNN achieved weighted F1-scores of 99.40% on CSE-CIC-IDS2018 and 95.77% on SUNE, corresponding to a difference of 3.63 percentage points despite substantial differences in network scale, attack categories, traffic protocols, and institutional context. FPR remained low across both datasets at 0.09 and 0.81%, respectively. These results suggest that ACAFS maintained stable feature relevance and detection capability across different network environments without dataset-specific manual tuning. This consistency is particularly important for intrusion detection deployment in heterogeneous institutional environments where traffic characteristics may vary substantially.

### 3.8 Comparative analysis with related works

Table 12 compares the proposed ACAFS framework with recent intrusion detection studies evaluated on CSE-CIC-IDS2018. The proposed method achieved an accuracy of 99.39% and weighted F1-score of 99.40%, remaining competitive with all compared approaches. The strong performance observed across both overall and per-class metrics may be attributed to the class-aware feature selection strategy, which preserves discriminative information for minority attack categories while reducing redundant dimensions. The low FPR is particularly important for operational NIDS deployment, where excessive false alarms can reduce analyst efficiency and trust in the detection system.

**Table 12:** Comparative analysis with related works.

Author	Accuracy	Precision	Recall	F1-Score
Bakro et al. (2022)	98.09	96.24	88.53	91.87
Alzughaibi and El Khediri (2023)	98.41	99.55	98.85	99.20
Harini et al. (2023)	98.3	98.1	86.42	98.42
Henry et al. (2023)	98.73	—	—	—
Hu et al. (2024)	99.06	99.31	99.28	99.28
Proposed	99.39	99.43	99.39	99.40

In addition to comparisons with related intrusion detection studies, ACAFS was evaluated against representative feature selection baselines under identical experimental conditions. The results presented in Table 13 show that ACAFS achieved the highest F1-score of 99.40%, lowest FPR of 0.09%, and strongest minority-class recall while selecting the smallest feature subset of 22 features. Compared with mRMR and RFECV, ACAFS consistently improved both overall classification performance and minority-class detection performance, demonstrating that the proposed class-aware feature selection strategy preserves discriminative information more effectively than conventional feature selection approaches under severe class imbalance.

**Table 13:** Comparison of ACAFS with representative feature selection methods.

Method	Feature	Accuracy	F1-score	FPR	Web_Recall	Inf_Recall
All features	74	98.62	98.79	0.19	93.84	95.62
mRMR	30	98.91	98.96	0.16	95.41	96.38
RFECV	26	99.01	99.04	0.14	96.21	97.13
ACAFS	22	99.39	99.40	0.09	98.56	98.59

Direct comparison across studies should be interpreted carefully, as differences in pre-processing strategies, dataset versions, class consolidation approaches, and evaluation protocols may influence reported performance. In addition, high overall accuracy alone may not adequately reflect minority-class detection capability under severe class imbalance, highlighting the importance of per-class evaluation metrics.

## 4 Discussion

This study evaluated the ACAFS framework across two heterogeneous network datasets CSE-CIC-IDS2018 and SUNE under identical pre-processing, balancing, training, and evaluation conditions. The experimental results confirm that adaptive and class-aware feature selection substantially improves intrusion detection performance across heterogeneous network environments, particularly for minority attack categories that are systematically overlooked by conventional global feature scoring approaches. The strong performance achieved on both CSE-CIC-IDS2018 and SUNE without dataset-specific parameter adjustment suggests that the permutation-based adaptive thresholding mechanism effectively captures dataset-specific statistical characteristics, eliminating the need for manual feature count specification. These findings further indicate that feature relevance in imbalanced intrusion detection should not be evaluated solely through global statistical importance, as minority-class discriminative information may otherwise be suppressed by dominant traffic classes (Al-Sarem et al., 2021; Chen et al., 2024; Gul et al., 2025).

A key finding of this study is the ability of ACAFS to preserve discriminative information for minority attack classes, which remains insufficiently addressed in many existing intrusion detection approaches. Classical filter-based methods evaluate features independently and do not incorporate class-specific information (Min and Wei, 2023), while many deep learning approaches rely on implicit feature learning without ensuring that minority-relevant features are preserved (Alzughaibi and El Khediri, 2023). Even approaches that explicitly address class imbalance through sampling strategies may still fail to achieve strong minority-class performance when feature selection is not class-aware (Yin et al., 2023; Al-Sarem et al., 2021). The proposed ACAFS framework addresses this limitation by integrating class-aware Composite Mutual Information scoring, ensuring that features relevant to minority attack categories are retained during the selection process. This design directly contributes to the strong detection performance observed for minority classes, including an Infiltration F1-score of 0.9926 and Web_Attacks recall of 0.9859. These results highlight that effective intrusion detection requires not only high overall accuracy but also balanced performance across all classes. The observed performance further indicates that the effectiveness of ACAFS-selected features may vary across different learning architectures.

The superior performance of the Two-Stage CNN may be attributed to its hierarchical decomposition of the intrusion detection task, allowing the Gate CNN to first separate benign and malicious traffic before fine-grained attack classification is performed by the Attack CNN. This staged learning strategy likely reduced class competition during optimization and improved sensitivity to minority attack categories. In contrast, the comparatively lower performance of TabTransformer on SUNE may reflect the increased variability and heterogeneity of institutional network traffic, which can limit the effectiveness of attention-based feature interaction learning under smaller feature spaces.

The two-stage design of ACAFS further enhances its effectiveness by combining statistical relevance with empirical validation. The permutation-based adaptive thresholding stage removes features whose relevance is statistically indistinguishable from noise, while the XGBoost refinement stage ensures that retained features contribute meaningfully to predictive performance. This dual-evidence selection mechanism reduces the likelihood of preserving redundant dimensions while maintaining features that are informative for both majority and minority attack categories. This is particularly important in high-dimensional network traffic datasets, where redundant flow attributes may obscure minority-class patterns and reduce model generalization (Al-Sarem et al., 2021; Chen et al., 2024).

In addition to classification performance, the reduced feature subset provides operational advantages for real-time deployment by lowering computational complexity, reducing training cost, and improving inference efficiency. Furthermore, the interpretability of the selected features improves understanding of model decisions and facilitates integration into operational security environments.

The consistent performance of ACAFS across CSE-CIC-IDS2018 and SUNE datasets demonstrates its robustness across heterogeneous network environments. The ability to achieve strong results without dataset-specific tuning highlights the generalizability of the proposed framework. However, one limitation observed is the lower precision for Web_Attacks despite its high recall. This precision–recall trade-off is likely attributable to behavioral overlap between Web-based attacks and legitimate HTTP/HTTPS traffic, which share similar flow-level characteristics and increase the likelihood of false positive predictions. Similarly, Probe traffic represented the most challenging attack category on the SUNE dataset. Analysis of the confusion matrix indicates that a substantial proportion of Probe samples were misclassified as U2R traffic, suggesting overlap between their flow-level behavioral characteristics. These findings indicate that minority attack detection remains strongly influenced by behavioral overlap and traffic variability, and suggest that further improvements may require additional discriminative information beyond flow-level statistics, such as temporal behavioral patterns or payload-based features.

## 5 Conclusion and future work

This study addressed key limitations of existing feature selection approaches for network intrusion detection, particularly their reliance on global feature importance and lack of class-aware mechanisms under severe class imbalance. To address these limitations, the Adaptive Class-Aware Feature Selection (ACAFS) framework was proposed, combining permutation-based adaptive thresholding, Composite Mutual Information scoring, and XGBoost-based refinement.

Experimental results on the CSE-CIC-IDS2018 dataset demonstrate that the proposed framework achieves 99.39% accuracy and 99.40% F1-score while reducing the feature space by 70.3%. More importantly, ACAFS improves detection performance for minority attack classes, achieving an Infiltration F1-score of 0.9926 and a Web_Attacks recall of 0.9859. These results demonstrate improved detection capability for rare and critical attack categories compared to many existing approaches that primarily emphasize overall classification performance.

The findings suggest that effective intrusion detection depends not only on classifier design but also on the construction of the feature space. By integrating adaptive and class-aware feature selection, the proposed framework highlights the relationship between dimensionality reduction, minority-class sensitivity, and practical intrusion detection deployment. In addition, the consistent performance observed across heterogeneous datasets suggests that ACAFS demonstrates consistent generalization across different network environments without dataset-specific manual tuning.

Despite these strengths, the study has limitations. The evaluation was conducted in an offline setting and on a limited number of datasets, which may not fully capture evolving real-world traffic behavior and attack diversity. Future work will prioritize three main directions motivated by the findings and limitations of this study. First, the ACAFS framework will be extended to real-time streaming environments, where the permutation-based adaptive thresholding mechanism must be adapted to handle continuous and evolving traffic distributions without recomputing the full null distribution from scratch, which is particularly important for operational NIDS deployment where network traffic characteristics evolve over time. Second, the behavioral overlap observed between certain attack categories and legitimate network activity reflected in the lower Web_Attacks precision and Probe recall on SUNE motivates future exploration of payload-based inspection, temporal behavioral sequences, and graph-based communication pattern features as complementary inputs to flow-level statistics to improve class separability for behaviorally similar attack categories. Third, future research will evaluate the robustness of the proposed framework under adversarial conditions where attackers deliberately manipulate traffic features to evade detection, and will explore class-specific modeling strategies and adaptive probability calibration mechanisms to further improve detection sensitivity for challenging minority attack categories such as Web_Attacks and Probe traffic.

Overall, the findings demonstrate the potential of adaptive and class-aware feature selection for developing efficient and minority-aware intrusion detection systems across heterogeneous network environments.

## Data Availability

The raw data supporting the conclusions of this article will be made available by the authors, without undue reservation.
